# Gene expression signatures modulated by epidermal growth factor receptor activation and their relationship to cetuximab resistance in head and neck squamous cell carcinoma

**DOI:** 10.1186/1471-2164-13-160

**Published:** 2012-05-01

**Authors:** Elana J Fertig, Qing Ren, Haixia Cheng, Hiromitsu Hatakeyama, Adam P Dicker, Ulrich Rodeck, Michael Considine, Michael F Ochs, Christine H Chung

**Affiliations:** 1 Department of Oncology, Sidney Kimmel Comprehensive Cancer Center, School of Medicine, Johns Hopkins University, Baltimore, MD, USA; 2 Department of Radiation Oncology, Kimmel Cancer Center, Thomas Jefferson University, Philadelphia, PA, USA; 3 Department of Otolaryngology, Hokkaido University, Sapporo, Hokkaido, Japan; 4 Department of Pharmacology and Experimental Therapeutics, Kimmel Cancer Center, Thomas Jefferson University, Philadelphia, PA, USA; 5 Department of Dermatology and Cutaneous Biology, Thomas Jefferson University, Philadelphia, PA, USA; 6 Department of Health Science Informatics, School of Medicine, Johns Hopkins University, Baltimore, MD, USA

## Abstract

**Background:**

Aberrant activation of signaling pathways downstream of epidermal growth factor receptor (EGFR) has been hypothesized to be one of the mechanisms of cetuximab (a monoclonal antibody against EGFR) resistance in head and neck squamous cell carcinoma (HNSCC). To infer relevant and specific pathway activation downstream of EGFR from gene expression in HNSCC, we generated gene expression signatures using immortalized keratinocytes (HaCaT) subjected to ligand stimulation and transfected with EGFR, RELA/p65, or HRAS^Val12D^.

**Results:**

The gene expression patterns that distinguished the HaCaT variants and conditions were inferred using the Markov chain Monte Carlo (MCMC) matrix factorization algorithm Coordinated Gene Activity in Pattern Sets (CoGAPS). This approach inferred gene expression signatures with greater relevance to cell signaling pathway activation than the expression signatures inferred with standard linear models. Furthermore, the pathway signature generated using HaCaT-HRAS^Val12D^ further associated with the cetuximab treatment response in isogenic cetuximab-sensitive (UMSCC1) and -resistant (1CC8) cell lines.

**Conclusions:**

Our data suggest that the CoGAPS algorithm can generate gene expression signatures that are pertinent to downstream effects of receptor signaling pathway activation and potentially be useful in modeling resistance mechanisms to targeted therapies.

## Background

Aberrant signal transduction pathways induce and maintain many cancers [1-3]. Therefore, targeted therapeutics blocking this aberrant cellular signaling activity can impede malignant progression. However, the clinical success of targeted agents relies on accurate identification of the relative contribution of the targeted pathway to the malignancy prior to treatment. For example, EGFR overexpression is associated with poor prognosis of head and neck squamous cell carcinoma (HNSCC) leading to adoption of EGFR-targeted agents including cetuximab, a monoclonal antibody against EGFR, for clinical management [4-7]. Although many HNSCC patients benefit from cetuximab treatment, a majority of patients do not respond or eventually develop acquired resistance after the initial clinical response. Previous studies have implicated activation of epistatic signaling intermediaries downstream of EGFR activation in cetuximab resistance [8]. Thus, inference of aberrant pathway activity controlled by EGFR activation may shed light on molecular underpinnings of acquired cetuximab resistance in patients with HNSCC.

Gene expression profiles constitute an important tool to investigate and predict biochemical network activity in complex cellular systems such as human tumors. Standard class discovery techniques, such as hierarchical clustering [9] and singular value decomposition (SVD; [10]), can implicate gene expression activity common to subsets of samples. However, clustering algorithms are unable to account for the reuse of genes in diverse biological processes as is common in eukaryotic systems [11,12]. Moreover, algorithms such as SVD infer complex combinations of responses across all measured genes, without regard for the biochemical structure of cellular signaling networks. As a result, these inference techniques often obscure the relationship of the resulting genetic signatures to the specific cell signaling processes that are active in the measured system [13].

On the other hand, coordinated expression changes in a priori sets of downstream targets of pathway-activated transcription factors can implicate activity in specific signaling pathways [14-16]. These pathway-inference techniques predominately use statistical techniques that quantify the magnitude of class comparison statistics, notably t-statistics, in a priori gene sets relating to cell signaling relative to a background, null distribution (reviewed in [17]). Similar to clustering algorithms, co-regulation of individual genes by multiple pathways and transcription factors will bias these gene set-based analysis techniques [18]. Previous studies have shown that Markov chain Monte Carlo (MCMC) matrix factorization techniques, such as Bayesian Decomposition (BD; [11]) and Coordinated Gene Activity in Pattern Sets (CoGAPS; [19]), robustly infer gene expression patterns relating to transcription factor activity in cancers [13].

To generate pathway signatures for HNSCC that are well characterized at the molecular level, we use HaCaT keratinocytes and isogenic variants thereof. HaCaT cells are well characterized in reference to their biological and malignant properties [20-23]. Therefore, these HaCaT models provide a potential to identify oncogenic signatures related to signaling responses that are unencumbered by ‘background noise’ inherent to tumor tissues. The HaCaT cell lines have the further advantage as a model system because their genetic aberrations closely parallel early oncogenic events seen in HNSCC. Specifically, like HNSCC [24], the HaCaT lines represent a spontaneously immortalized aneuploid human keratinocyte cell line of monoclonal origin with increased telomerase activity [25], two mutant p53 alleles [26], and absence of p16^INK4a^ expression due to promoter hypermethylation [27].

Here we show that the MCMC matrix factorization algorithm CoGAPS infers gene expression patterns in HaCaT keratinocytes associated with modulation of the EGFR activity by forced expression, ligand stimulation, and pharmacological inhibition [28]. We relate these patterns directly to activity at the pathway level, in contrast to previous studies that have focused on transcription factor-level activity [13,29]. In addition, the CoGAPS inferred patterns predict molecular response to cetuximab treatment in an isogenic pair of HNSCC cell lines with varying cetuximab sensitivities.

## Results

### Compilation of protein-protein interaction data in the signaling pathways downstream of EGFR in HNSCC for pathway-level gene set inference

**Figure 1:**
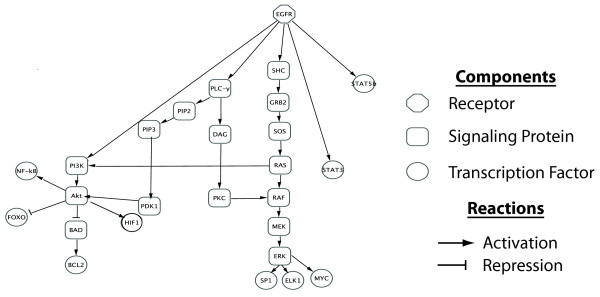
**EGFR signaling network in HNSCC.** Simplified diagram of the protein-protein interaction network downstream of EGFR in HNSCC adapted from [30,31].

A number of molecular interactions triggered by EGFR activation have been characterized in HNSCC. Figure 1 summarizes the corresponding pathway diagram of canonical protein-protein interactions in EGFR signaling in HNSCC compiled from reviews by [30,31]. This diagram displays that many of the EGFR-dependent signaling events culminate in activation of distinct subsets of transcription factors (Additional file 1: Additional file 6: Table S 1). Although not directly downstream of EGFR, Addional file 6: Table S 1 also shows transcription factors activated by the Notch and TGF-β pathways. These pathways are included in our analyses because they intersect with EGFR-dependent signaling events and have previously been implicated in HNSCC biology [32-35]. The pathway-activated transcription factors and associated targets listed in Additional file 6: Table S 1 were used to infer specific pathway activity from Affymetrix microarray measurements of HNSCC using the gene set statistics from the CoGAPS algorithm (eqs. 2 and 3) and from linear models [36] as described in the methods section.

### CoGAPS reveals transcriptional responses to EGFR pathway modulation in the isogenic HaCaT model system reflected in their gene expression changes

Isogenic HaCaT variants overexpressing either wild-type EGFR or the transcription factor NF-kappa-B p65 subunit (p65), or mutant HRAS have previously been described by [23,28,37,38]. In order to delineate specific targets resulting from activating these pathways, we measured gene expression patterns associated with increased expression of wild-type EGFR (HaCaT-EGFR^WT^) and p65 (HaCaT-p65^WT^) with and without serum and ligand stimulation (EGF and TNF-α, for 4 h or 8 h) and from constitutive activation of HRAS imparted by an activating mutation (HaCaT-HRAS^Val12D^) concurrently with the pathway modulation demonstrated in [28]. Table 1 further summarizes the experimental design for these gene expression data.

**Table 1:** HaCaT experimental design

**Over-expression**	**Media**	**Treatment**	**Replicates**
none	serum	none	3
vector	serum	none	3
vector	none	none	4
vector	EGF	none	4
vector	TNF	none	3
HRAS^Val12D^	serum	none	3
p65^WT^	serum	none	3
p65^WT^	none	none	3
p65^WT^	TNF	none	5
EGFR^WT^	serum	none	5
EGFR^WT^	none	none	7
EGFR^WT^	EGF	none	7

Summary of the experimental conditions in the HaCaT cell line expression experiments, provided in further detail in Supplemental File 5.

CoGAPS infers the relative presence of patterns in gene expression (columns of the matrix **A**) across samples measured (rows of **P**) from a matrix of microarray data **D** with N genes (rows) and M samples (columns). Specifically, it uses the MCMC algorithm described in the Methods section to find the optimal, sparse non-negative matrices **A** and **P** according to the equation

(1) $$D\sim N\left(AP,\Sigma \right)$$

where N represents the normal distribution on each element of the matrix multiplication **AP** and the i^th^ row and j^th^ column of the matrix **Σ** is the standard deviation of gene expression for the i^th^ gene and j^th^ sample. The resulting signals that are common to subsets of the samples are summarized in the rows of the pattern matrix **P**, with related gene expression patterns in the corresponding columns of **A**. In contrast to standard techniques class-comparison algorithms that strictly infer differential expression between sets of samples, the CoGAPS analysis infers predominant signals from the gene expression data in an unsupervised analysis. As a result, CoGAPS can capture degrees of gene expression activity that are common to various sample classes. A further advantage of CoGAPS over standard class-comparison or clustering algorithms is its ability to infer activity in subsets of genes concurrently affected by the diverse biological processes in each sample type [12,19,29].

**Figure 2:**
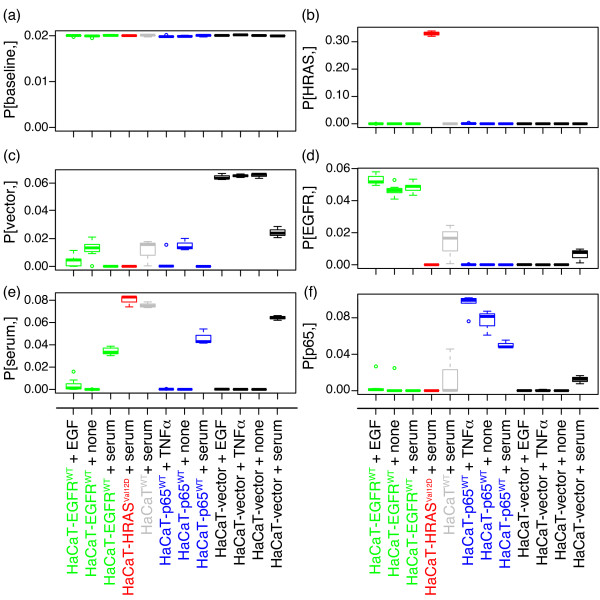
**CoGAPS inferred gene expression patterns.** Box plot of six gene expression patterns inferred from the HaCaT gene expression data for samples in Table 1. Plotted values are normalized to sum to one across all samples and are averaged across three applications of the CoGAPS algorithm. All results for HaCaT-EGFR^WT^ are colored in green, HaCaT-HRAS^Val12D^ in red, HaCaT-EGFR^WT^ in grey, HaCaT-p65^WT^ in blue, and HaCaT-vector in black. The y-axis is labeled according to the row of the inferred **P** matrix plotted in each panel. Specifically, (a) contains the pattern attributed to the baseline HaCaT activity, (b) attributed to HaCaT-HRAS^Val12D^, (c) HaCaT-vector control, (d) HaCaT-EGFR^WT^, (e) serum, and (f) HaCaT-p65^WT^.

When decomposing the data from the HaCaT model system according to eq. 1, CoGAPS infers six patterns. These patterns are robust in that they are conserved across three separate simulations of the CoGAPS algorithm (Additional file 1: Figure S 1). Therefore, all subsequent analyses are performed for the average patterns inferred from these simulations. Although the CoGAPS analysis is unsupervised, the six inferred patterns plotted in Figure 2 separate samples based on the experimental conditions summarized in Table 1. Specifically, CoGAPS infers patterns that relate to the molecular changes introduced in the isogenic HaCaT model system; *EGFR*^*WT*^, *p65*^*WT*^, *HRAS*^*Val12D*^ and empty vector control. Likewise, CoGAPS infers an additional pattern that reflects pathway activity resulting from the exposure of cell lines to fetal calf serum. When serum starved, both HaCaT-EGFR^WT^ and HaCaT-p65^WT^ show weak upregulation in the pattern associated with the control HaCaT-vector (Figure 2c; one sided p-value of 0.001 for HaCaT-EGFR^WT^ and 0.013 for HaCaT-p65^WT^). Moreover, incorporating the EGF ligand in the media for HaCaT-EGFR^WT^ (Figure 2d) and the TNF-α ligand for HaCaT-p65^WT^ (Figure 2f) moderately increases the association of both cell lines to their respective patterns over their serum starved counterparts (one-sided p-value of 0.002 for HaCaT-EGFR^WT^ and 0.065 for HaCaT-p65^WT^).

**Figure 3:**
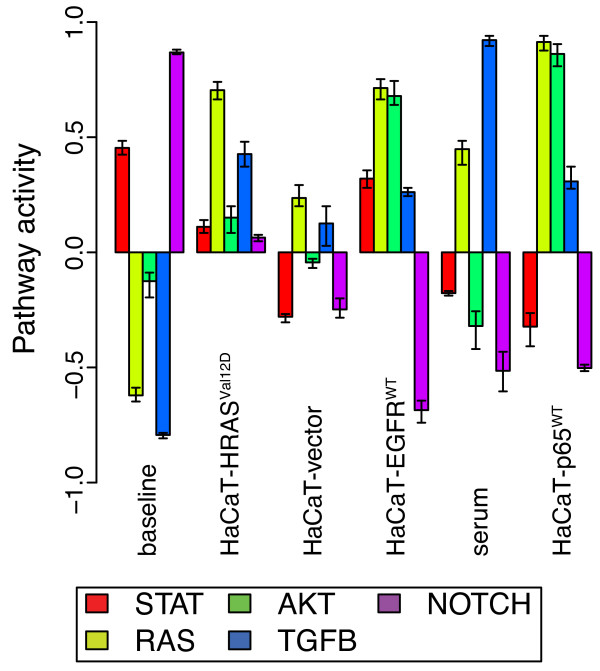
**HaCaT expression CoGAPS gene set statistics.** Gene set statistics for pathway activity of the HaCaT expression data calculated from eq. 3 averaged across three CoGAPS simulations. Error bars indicate minimum and maximum values of the pathway activity statistic in each of the three CoGAPS simulations. Bars are grouped and labeled according to the dominant experimental condition to which inferred CoGAPS patterns correspond. Within each block, statistics are provided for the STAT, RAS, AKT, TGFB, and Notch pathways (left to right) colored according to the figure legend.

In accordance with eq. 1, the inferred patterns from the rows of **P** can be linked to signatures of gene expression in the columns of the amplitude matrix **A** to implicate relative gene expression in subsets of samples. We apply the *Z*-score gene-set statistic of [13] (eqs. 2 and 3 of the Methods section) to infer pattern-specific pathway activation (repression) from over- (under-) representation of large magnitude elements in the A matrix for genes that are targets of transcription factors. Whereas [13] limited application of this statistic to targets of transcription factors, Figure 3 displays estimates of activation and repression of pathways and Additional file 2: Figure S 2 the statistics for component transcription factors (listed in Additional file 1: Table S 1) estimated with eq. 3 for the HaCaT data. Therefore, in this application a pathway is inferred to be either up or down regulated if its targets have a correspondingly larger or smaller *Z*-score in elements of the **A** matrix relative to a random selection of genes. By basing this statistic on the relative standard-deviation adjusted magnitude in elements of **A**, CoGAPS directly infers pathway activity that is active in each pattern inferred (row of **P**). Therefore, CoGAPS provides a direct implication of relative pathway activity in samples in contrast to the inference limited to differential pathway activity in gene set statistics in standard class-comparison algorithms. Similar to the inferred patterns, these pathway and transcription factor level statistics are robust across CoGAPS simulations (Figure 3 and S 3).

In analyzing the CoGAPS estimated pathway activity (Figure 3), we observe the expected global upregulation of STAT, RAS, and AKT pathways in HaCaT-EGFR^WT^, upregulation of RAS in HaCaT-HRAS^Val12D^, and upregulation of AKT resulting in HaCaT-p65^WT^. We also observe unexpected weak activation of TGF-β in HaCaT-EGFR^WT^ and of RAS and TGF-β in HaCaT-p65^WT^. These unexpected signals are likely due to pathway cross talk and the observed weak upregulation of RAS and TGF-β pathways in HaCaT-vector control. The presence of serum also enhances this upregulation of RAS and TGF-β. The final pattern is consistent across samples and reveals a global upregulation of Notch and STAT pathways in the HaCaT cell lines.

### Comparison of gene expression signatures inferred in CoGAPS and linear models

We note that the six CoGAPS patterns are inferred without any prior information about the experimental conditions in Table 1, whereas standard linear models of pathway response require information about the pertinent experimental conditions. As a result, linear models formed based upon the experimental conditions in the HaCaT forced expression experiments may overfit expression changes due to treatment and stimulation conditions. Moreover, they would be unable to account for relative changes in the magnitude of expression across comparable samples encoded in the relative magnitude of rows of **P** (Figure 2).

**Figure 4:**
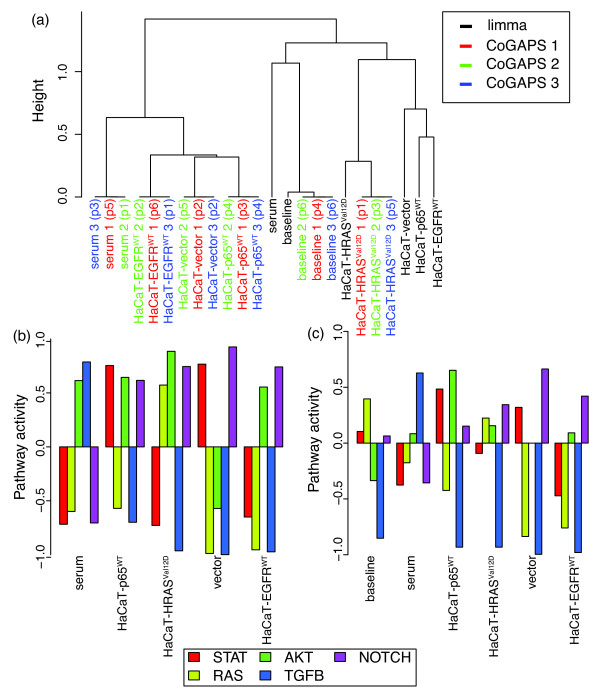
**Comparison of CoGAPS and linear models.** (a) Clustering diagram to compare the gene signatures in the columns of **A** from eq. 1 in each of three CoGAPS simulations (simulation 1 in red, simulation 2 in green, and simulation 3 in blue) to the signatures inferred from a limma linear model using the same design matrix (black). (b) and (c) display gene set statistics for pathway activity resulting from the limma estimated linear model for STAT, RAS, AKT, TGFB, and Notch (left to right within each group) colored according to the bottom figure legend. (b) Uses the standard gene set test from limma, rescaled according to eq. 4. (c) Uses the permutation-based gene set statistic adapted for a linear model from eqs. 2 and 3 described in the methods section.

For comparison, we formulate a linear model that accounts for the forced overexpression conditions and presence or absence of serum based upon the inferred CoGAPS patterns. In Figure 4a, we observe that the expression patterns inferred for the linear model across all HaCaT samples (baseline), all serum treated samples in HaCaT, and all HaCaT-HRAS^Val12D^ samples are most similar to the corresponding CoGAPS patterns. However, the patterns associated with known molecular variables in HaCaT cells cluster separately from the CoGAPS estimated patterns. These patterns also show a larger difference in their clustering than the patterns of molecular variables inferred from CoGAPS.

Figure 4b and 4c show the pathway statistics from the linear model gene expression signatures using standard gene set tests and a test analogous to CoGAPS, respectively, as described in the Methods section. In contrast to CoGAPS (Figure 3), the gene set statistics from the linear model (Figure 4b) show strong upregulation of only the AKT and Notch pathways in both the EGFR and RAS pathways, with only weak upregulation of RAS, which would be predicted from the experimental conditions. While the linear model reveals expected upregulation of the AKT pathway in HaCaT-p65^WT^, it also infers unexpected upregulation of Notch and strong upregulation of the STAT pathway. This latter STAT upregulation due to p65 overexpression is inconsistent with the structure of EGFR protein-protein interactions (Figure 1), in which p65 is far downstream of STAT. In further contrast to the CoGAPS gene expression patterns, the linear model does not infer expected STAT pathway upregulation in the HaCaT-HRAS^Val12D^ or HaCaT-EGFR^WT^ data. Similar to CoGAPS, the linear model infers a strong upregulation of TGF-β from the introduction of serum. Unlike CoGAPS, the linear model also predicts that serum strongly upregulates signaling in the RAS pathway. The signaling patterns are largely similar using the CoGAPS-based permutation statistics from eq. 4 in Figure 4c. In this case, the linear model statistics do infer a weak upregulation of RAS in HaCaT-HRAS^Val12D^. However, unlike the gene expression patterns from CoGAPS, the linear model for HaCaT-EGFR^WT^ still does not infer the expected upregulation of the STAT or RAS pathways.

### Gene expression signatures from CoGAPS distinguish pathway-level response in isogenic cetuximab sensitive and resistant HNSCC cell lines

**Figure 5:**
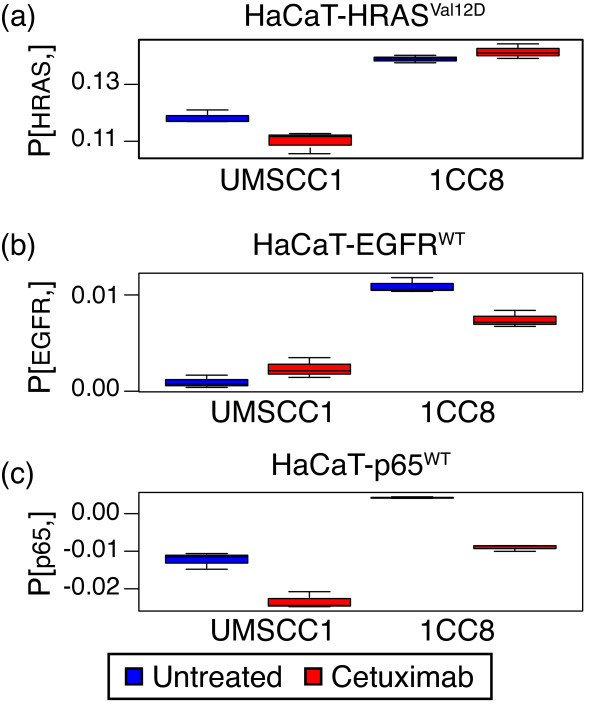
**CoGAPS signature in UMSCC1 and 1CC8 cell lines.** Box plot of the **P** matrix resulting from projecting the CoGAPS inferred gene expression signatures for (a) HaCaT-HRAS^Val12D^, (b) HaCaT-EGFR^WT^, and (c) HaCaT-p65^WT^ onto gene expression data for UMSCC1 (left group) and 1CC8 cell lines (right group). Results for data treated with cetuximab are colored in red (right in each group) and untreated are colored in blue (left in each group).

We project the three HaCaT signaling patterns averaged across three CoGAPS simulations and associated with the HaCaT forced over-expression (HaCaT-HRAS^Val12D^, HaCaT-EGFR^WT^, and HaCaT-p65^WT^) onto the gene expression data for HNSCC cell lines UMSCC1 and 1CC8. As previously described, this pair of cell lines are isogenic sensitive and resistant HNSCC lines, respectively [8]. Thus, we can infer whether the signaling-related gene expression signatures elucidate the molecular mechanisms underlying cetuximab resistance in this pair of cell lines to potentially model cetuximab resistance in HNSCC. Figure 5 summarizes the relative CoGAPS inferred signature activity in UMSCC1 with and without cetuximab treatment, and 1CC8 with and without cetuximab treatment. The signatures associated with modulation of EGFR^WT^, p65^WT^ and HRAS^Val12D^ are all significantly larger in 1CC8 than UMSCC1 (p-value of 7x10^-4^ for the HaCaT-HRAS^Val12D^ signature, 8x10^-5^ for the HaCaT-EGFR^WT^ signature, and 5x10^-3^ for HaCaT-p65^WT^ signature). Treatment with cetuximab in the sensitive cell line UMSCC1 further reduces the activity in HaCaT-HRAS^Val12D^ (p-value 0.042) and HaCaT-p65^WT^ (p-value 0.004), but upregulated the signature associated with HaCaT-EGFR^WT^ (p-value 0.13). A similar trend is observed for 1CC8 (p-value of 0.007 for HaCaT-EGFR^WT^ and 0.0008 for HaCaT-p65^WT^). However, whereas treatment with cetuximab reduces the HaCaT-HRAS^Val12D^ signature in the sensitive cell line UMSCC1, it has no effect on treatment in the resistant cell line 1CC8 (p-value of 0.23).

## Discussion

CoGAPS demonstrated improved ability to correctly infer patterns with even subtle transcriptional differences because this algorithm can accurately account for gene re-use [12,19,39]. In contrast to linear models, CoGAPS seeks gene expression signatures with minimal structure. As a result, the signatures inferred with CoGAPS are more similar than those inferred with linear models. Therefore, these CoGAPS expression signatures reveal degrees of activation of the pathways downstream of EGFR due to pathway cross-talk, unlike the standard gene set test or permutation gene set analysis for linear models. For example, only the CoGAPS analysis can detect the modest RAS and STAT signals associated with forced expression of EGFR and HRAS in HaCaT cells. Moreover, the sparsity in the CoGAPS prior ensures that gene expression amplitude is assigned only to pertinent patterns. As a result, while the linear model assigns Notch activity to each of the forced expression signatures, CoGAPS correctly assigns this global signature to the background term.

This type of statistical modeling can delineate the key pathway activity of relevance to the development of targeted agents in genetically heterogeneous HNSCC. In our previous molecular characterization of HNSCC based on gene expression profiling, we have shown that there are at least four subtypes of HNSCC and these subtypes have prognostic implications reflecting the biological heterogeneity [40]. In addition, recent completion of whole exome sequencing of HNSCC has shown that tobacco-related HNSCC contain average of 20.6 mutations per tumor and many of the mutated genes are tumor suppressors that cannot be easily targeted [34,35]. Furthermore, many of these mutated genes are regulated in contextual manner meaning that one mutation of a gene in a tumor may result in a different phenotype depending on the genetic context determined by other co-existing mutations or deregulations. The analyses of global gene expression signature will potentially yield the dominant pathways that result from lack of tumor suppressor or contextual regulated genes, and allow exploitation as therapeutic targets or biomarkers of clinical outcome. It is also a potentially valuable tool of determining the on- and off-target effects of targeted agents and hypothesis generating approach to unravel the mechanism of resistance by unbiased examination of global changes in the inter-connected pathways induced by inhibition of the targeted pathway. Due to the small sample size, we could not infer accurate expression signatures from the pharmacological inhibition of EGFR, MEK or PI3K in our model system. However, additional studies to optimize the experimental design and to further validate the model for utilization in experimental therapeutics are in progress.

In this study, the inferred CoGAPS gene expression signatures implicate signaling processes in the HNSCC system that they model. For example, the gene expression signature related to constitutive activation of the RAS pathway in the HaCaT-HRAS^Val12D^ distinguishes the transcriptional profile of UMSCC1 and 1CC8 and their relative transcriptional response to cetuximab treatment. This observation suggests that over-activation of the RAS pathway cannot be repressed by cetuximab in resistant HNSCC cell lines. While HRAS mutations are found in HNSCC [34], the UMSCC1 and 1CC8 cell lines are HRAS wild type. Therefore, this aberrant activity in the RAS pathway that our algorithm inferred for 1CC8 is consistent with activation of the wild type RAS pathway in cetuximab resistance. One possible mechanism for this activation would be the compensatory pathway activity proposed in [8]. Further studies to validate the mechanisms underlying these are currently ongoing.

## Conclusions

This work demonstrates the versatility of the CoGAPS matrix factorization algorithm to infer biological signaling nodes and intermediaries as they relate to specific gene expression in immortalized HaCaT and transformed variants thereof. For example, upon stimulation/deregulation of EGFR activity, the algorithm successfully identified gene expression signatures consistent with known elements of the EGFR signaling network (Figure 1). In contrast to linear models, the CoGAPS algorithm performs pathway inference without a priori knowledge of the experimental conditions listed in Table 1. Pathway inference by CoGAPS was predictive across a heterogeneous set of experimental pathway manipulations, i.e., transcriptional responses triggered either by overexpression of EGFR, NF-kB/p65 or mutant HRAS, or those induced by addition of serum to culture media.

Similarly to the UMSCC1/1CC8 model, we hypothesize that the pathway-level gene expression signatures inferred from the HaCaT model with CoGAPS will implicate relevant molecular mechanisms in gene expression data from HNSCC patients in future studies. For example, the gene expression signature relating to constitutive RAS activation estimated from the HaCaT-HRAS^Val12D^ provides a potential biomarker to infer patient-specific cetuximab sensitivity and resistance prior to clinical treatment. Moreover, previous studies have also linked cetuximab resistance to increased AKT pathway activity in HNSCC cell lines with cetuximab resistance [8]. We hypothesize that application of the CoGAPS algorithm to future samples of HaCaT with forced expression of RAF and PI3K with activating mutations will distinguish whether the activation of the RAS pathway induces AKT pathway activity preferentially over MAPK pathway (Figure 1) in cetuximab resistance. When applied to tumor data, these additional CoGAPS inferred gene expression signatures would also provide candidate molecular targets for patients with predicted cetuximab resistance.

## Methods

### Inhibition and stimulation of EGFR signaling pathway in the HaCaT model system and HNSCC cell lines

HaCaT cells overexpressing EGFR (HaCaT-EGFR) were maintained in cell culture media (W489) as previously described [20,22]. The cells were seeded in 100 mm tissue culture plates in regular media until they reach 70-80 % confluency. After incubation with serum-free media for 12 hours, EGF or TNF-α (10 ng/ml, Sigma-Aldrich, St. Louis, MO) were then added for 4 or 8 hours before cells were harvested for total RNA isolation. The culture conditions, total RNA isolation and microarray experiments of SCC1 and 1CC8 HNSCC cell lines used in the study were previously published [8].

### Microarray data preprocessing

To ensure that the microarray measurements from the HaCaT samples and the UMSCC1/1CC8 cell lines are comparable, we normalize all microarray measurements using fRMA [41]. After fRMA normalization, clustering reveals an apparent batch effect from processing date in the HaCaT expression data (Additional file 3: Figure S 3a). We, therefore, fit a linear model with date and experimental conditions summarized in Table 1 using the lmFit function in the R Bioconductor package limma (Linear Models for Microarray Data; [36]). Additional file 3: Figure S 3b shows that samples correctly cluster by experimental condition after removing the modeled date effect using the clustering diagram generated with [42]. This batch corrected, normalized data will be used for subsequent analyses of the HaCaT cell lines. For robustness, the clustering and linear models were performed on all HaCaT samples, including eight samples that were treated with pharmacological agents. These latter samples were excluded from subsequent analyses due to an inability to infer accurate expression signatures from their small samples size. On the other hand, the UMSCC1 and 1CC8 samples are processed in the same batch, requiring no correction after fRMA normalization. All the data are MIAME compliant and the raw data for all samples have been deposited in Gene Expression Omnibus (HaCaT data GSE32975; SCC1 and 1CC8 data in GSE21483). All analyses are performed with software R using scripts provided as Additional file 7: Files S 1, Additional file 8: File S 2, Additional file 9: File S 3, Additional file 10: File S 4 and Additional file 11: File S 5.

### Pathway and transcription factor targets

We identify candidate transcription factor regulators for each probe of the Affymetrix U133 Plus 2.0 array from TRANSFAC using the Automated Sequence Annotation Pipelines (ASAP; [43]). The list used in these analyses was obtained and frozen on December 8, 2010 and provided as a supplemental file containing the ASAP results (Additional file 7: File S 2). Gene sets related to each pathway listed in Additional file 6: Table S 1are defined as the targets of each transcription factor identified as downstream to the pathway from Additional file 6: Table S 1.

We limit all subsequent analyses of HaCaT and UMSCC1/1CC8 cell line expression to only those probes that are annotated in TRANSFAC. We select a single probe for each gene to further avoid biases in gene set tests from using multiple probes for a single gene. Specifically, we retain the probe for each gene with the smallest p-value resulting from comparisons of the HaCaT experimental conditions using t-statistics moderated with empirical Bayes from the limma package [36].

### CoGAPS pattern inference and pathway analysis

CoGAPS factors the expression matrix **D** into amplitude (**A**) and pattern (**P**) matrices with *p* patterns according to the distribution in eq. 1. The posterior distribution for elements of each of these matrices are computed with an MCMC Gibbs sampler based upon the atomic prior of [44] and implemented in the Bioconductor package CoGAPS [19]. Here, the standard deviation in eq. 1 is given by $\Sigma_{i,j}=0.1D_{i,j}$ for each gene *i* and sample *j*, based upon the established, microarray error-model in [45] and previous applications of [13,29].

The quality of the CoGAPS fit is assessed through the *χ*^2^ fit of the posterior mean of **A** and **P** and identifiability of these matrices across MCMC simulations. Using these criterion, the optimal number of patterns *p* for the matrix factorization is the minimum number of patterns for which the *χ*^2^ reaches a minimal value and the inferred patterns persist across simulations. For the HaCaT expression data analyzed in this paper, the *χ*^2^value of the fit begins to plateau for 6 patterns (Additional file 4: Figure S 4), at which point multiple CoGAPS simulations obtain the same **A** and **P** matrices (Additional file 1: Figures S 1 and Additional file 2: S 2).

Comparing the posterior distribution of the amplitude matrix for a pattern (i.e., column of **A**) between genes in a gene set and in the background can indicate over- or under-expression of that gene set in a pattern. Based upon previous work from [13], we define a Z-score to quantify the enrichment of gene-set G with *R* targets in pattern *p* by

(2) $$Z_{G,p}=\frac{1}{R}\sum_{g\in G}\frac{A_{\mathrm{gp}}}{\sigma_{\mathrm{gp}}}\text{,}$$

where $A_{\mathrm{g,p}}$ and $\sigma_{\mathrm{g,p}}$ are the sample mean and variance, respectively, from the CoGAPS MCMC samples for gene (row) *g* and pattern (column) *p* amplitude. We compute p-values through a permutation test that compares the computed value of $Z_{G,p}$ to a null distribution obtained by the values of the statistic in eq. 2 resulting from selecting random sets of *R* genes. In contrast to [13], we apply this statistic to both targets of individual transcription factors and targets of sets of transcription factors regulated in the pathways (Additional file 6: Table S 1) to infer pathway level activity. For visualization, p-values computed from the statistic in eq. 2 are transformed as follows

(3) $$\tilde{p}=2p-1.$$

Therefore, a transformed $\tilde{p}$ value of −1 indicates underrepresentation of set G and +1 indicates over-representation. The associated pathway-level statistics in Figure 3 represent the mean of the statistic from eq. (3) across three CoGAPS simulations, with error bars representing the minimum and maximum values in each of these simulations.

Projecting the gene expression signatures in the columns of **A** onto additional samples can implicate the relative activity of inferred patterns in those samples. In this paper, the projection is implemented by solving the factorization in eq. 1 for the new data matrix where **A** is fixed as the average of the CoGAPS posterior mean for each of the three CoGAPS simulations performed. We estimate the patterns **P** associated with this amplitude matrix using the least-squares fit to the new data implemented with the lmFit function in the limma package [36]. Applying this projection to the original, HaCaT data reveals that the projection provides similar, albeit slightly nosier, estimates when compared to the CoGAPS posterior mean for **P** (Additional file 5: Figure S 5). This linear projection is, therefore, used to project the gene signatures inferred from the HaCaT data onto gene expression data from the HNSCC UMSCC1 and 1CC8 cell lines. Differences between the values of the pattern matrix **P** inferred through CoGAPS or these subsequent projections are quantified using p-values from a *t*-test between the groups of experimental conditions being compared.

### Linear models and gene set pathway-level analysis

In the results section, we compare the CoGAPS inferred expression signatures in the columns of the **A** matrix to expression signatures inferred by the coefficients of a linear model. In this linear model, we use the limma package [36] to fit the batch-corrected HaCaT data to a design matrix comparable to the **P** matrix inferred in CoGAPS by specifying a common intercept term, HaCaT cell type (HaCaT-EGFR^WT^, HaCaT-p65^WT^, HaCaT-HRAS^Val12D^, or HaCaT-vector), and presence or absence of serum. Contrasts are formulated for each of these six conditions to obtain coefficients for each condition comparable to the columns of the CoGAPS **A** matrix (Figure 4a). The gene set statistic in Figure 4b uses the standard limma package geneSetTest function to test the probability of activity inferred from the contrast-based, empirical Bayes t-statistics in the pathway and transcription factor level targets in Additional file 6: Table S 1. To make values comparable to the CoGAPS analysis, only the subset of probes analyzed in CoGAPS (i.e., in TRANSFAC and summarized by gene) are considered for the background of the gene set statistics. Similar to eq. 3, we rescale the resulting statistics for visualization by

(4) $${\tilde{p}}_{\text{lm}}=\{\begin{array}{cc} \log_{10}\left(p_{\text{down}}\right) & \text{if }p_{\text{down}}<p_{\text{up,}}\\ -\log_{10}\left(p_{\text{up}}\right) & \text{otherwise} \end{array}$$

where *P*_down_ and *P*_up_ are the p-values resulting from the geneSetTest function if the alternative hypothesis is specified as down or up regulated, respectively. The heatmap in Figure 4b then rescales these statistics across columns are 1 at the maximum value of ${\tilde{p}}_{\text{lm}}$ and −1 at the minimum value. For further comparison to the CoGAPS results, Figure 4c plots the transformed p-values resulting from the permutation-based CoGAPS gene set test in eqs. 2 and 3. To reflect the statistics of the linear model, the posterior estimate for the ratio $\frac{A_{\mathrm{g,p}}}{\sigma_{\mathrm{g,p}}}$ in eq. 2 is replaced with the estimated, empirical Bayes moderated t-statistics for each of the six conditions specified in the linear model.

## Author’s contributions

EJF, MC, and MFO were responsible for data analyses and manuscript preparation. QR, HH, and HC performed data collection and assisted with manuscript preparation. UR and AD were involved with study design, data analysis, and manuscript preparation. CHC was responsible for study design, data collection, data analysis, and manuscript preparation.

## Acknowledgements and Funding

The authors would like to acknowledge funding from the following sources: EJF: K25 (CA141053), pilot project from the Sidney Kimmel Cancer Center of Johns Hopkins University Head and Neck SPORE; AD: funding from RTOG; UR: Commonwealth of Pennsylvania through the American College of Radiology and seed grant from TRP-RTOG; MFO: R21 (LM009382); CHC: R01 (DE017982) and Damon Runyon Clinical Investigator Award (CI-28-05).

## Supplementary Material

Additional file 3 **Figure S3. **Clustering of HaCaT expression data after fRMA colored by date (a). Analogous clustering after batch correction in (b). Click here for file

Additional file 7 **File S1. CompleteAnalysis.R:** R script used for to generate all analyses and figures. Click here for file

Additional file 8 **File S2. TF2Gene_2010.R:** R script encoding a list of transcription factor targets in TRANSFAC from ASAP. Click here for file

Additional file 9 **File S3. PathwayHeatmap.R:** Support script used to generate pathway-level heatmaps in Figures S1 and S3. Click here for file

Additional file 10 **File S4. ExperimentalDescription.txt:** Detailed annotation of experimental conditions summarized in Table 1. Click here for file

Additional file 11 **File S5. SCC11CC8Annot.txt:** Detailed annotation of the experimental conditions in the UMSCC1 and 1CC8 cell line expression datasets. Click here for file

Additional file 1 **Figure S1.**Box plot of six gene expression patterns inferred from the HaCaT gene expression data for each of the three CoGAPS simulations (pages 1–3) for the samples in Table 1. Plotted values are normalized to sum to one across all samples. All results for HaCaT-EGFR^WT^ are colored in green, HaCaT-HRAS^Val12D^ in red, HaCaT-EGFR^WT^ in grey, HaCaT-p65^WT^ in blue, and HaCaT-vector in black. The y-axis is labeled according to the row of the inferred **P** matrix plotted in each panel. Specifically, (a) contains the pattern attributed to the baseline HaCaT activity, (b) attributed to HaCaT-HRAS^Val12D^, (c) HaCaT-vector, (d) HaCaT-EGFR^WT^, (e) serum, and (f) HaCaT-p65^WT^. Click here for file

Additional file 2 **Figure S2.**Gene set statistics of the HaCaT expression data calculated from eq. 3 for each of the three CoGAPS simulations from blue for significantly downregulated to yellow for significantly upregulated according to the color bar. Columns are labeled according to the dominant experimental condition to which inferred CoGAPS patterns correspond and colored as indicated in the column color legend (red for the first CoGAPS simulation, green the second, and blue the third). The top set of statistics represents the gene set statistics computed at a pathway level. Colors along rows indicate the pathway for which activation statistics are calculated as indicated in the row color legend. The lower set of statistics represents the gene set statistics computed for the transcription factors activated by the pathway also indicated by colors in the rows associated with the pathway to which the transcription factor was assigned and indicated by the color code on the left. Click here for file

Additional file 4 **Figure S4.** χ^2^ fit from CoGAPS as a function of the number of patterns used in the matrix factorization for eq. 1. Click here for file

Additional file 6 **Table S1. PathwayTableS1.txt:** List of targets of transcription factors annotated to each pathway for pathway-level and transcription factor-level gene set analyses. Click here for file

Additional file 5 **Figure S5.** Heatmap comparing patterns inferred in CoGAPS as plotted Figure S2 (filled boxes on rows) to patterns that would be inferred from projecting expression patterns as described in the methods (open boxes on rows) colored according to the row figure legend. As indicated in the row figure legend, patterns are plotted for each of three CoGAPS simulations, colored in red (simulation 1), green (simulation 2), and blue (simulation 3) along the rows. The bars across the columns indicate media and forced expression conditions, colored according to the figure legend. Shading of these bars indicates media (white for serum starved, grey for serum, green for EGF, and blue for TNFα) while borders indicate forced expression (grey for HaCaT^WT^**,** black for HaCaT-vector, green for HaCaT-EGFR^WT^, blue for HaCaT-p65^WT^, and red for HaCaT-HRAS^Val12D^). Click here for file
