# Supplementary figures and images for: Gene expression signatures modulated by epidermal growth factor receptor activation and their relationship to cetuximab resistance in head and neck squamous cell carcinoma
Source: BMC Genomics. 2012 May 1;13:160. doi: 10.1186/1471-2164-13-160 (PMC3460736; doi:10.1186/1471-2164-13-160)

(a)

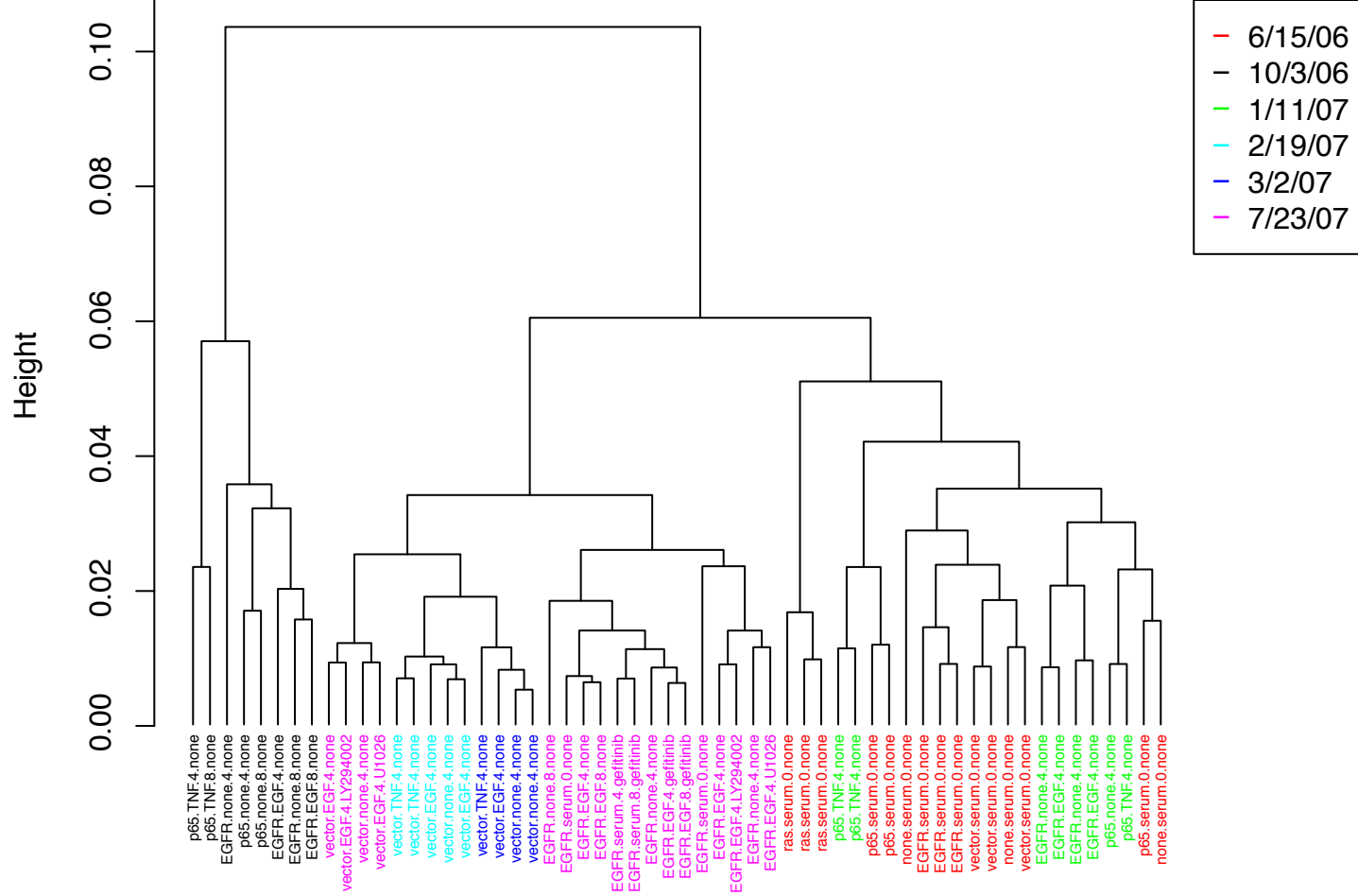

(b)

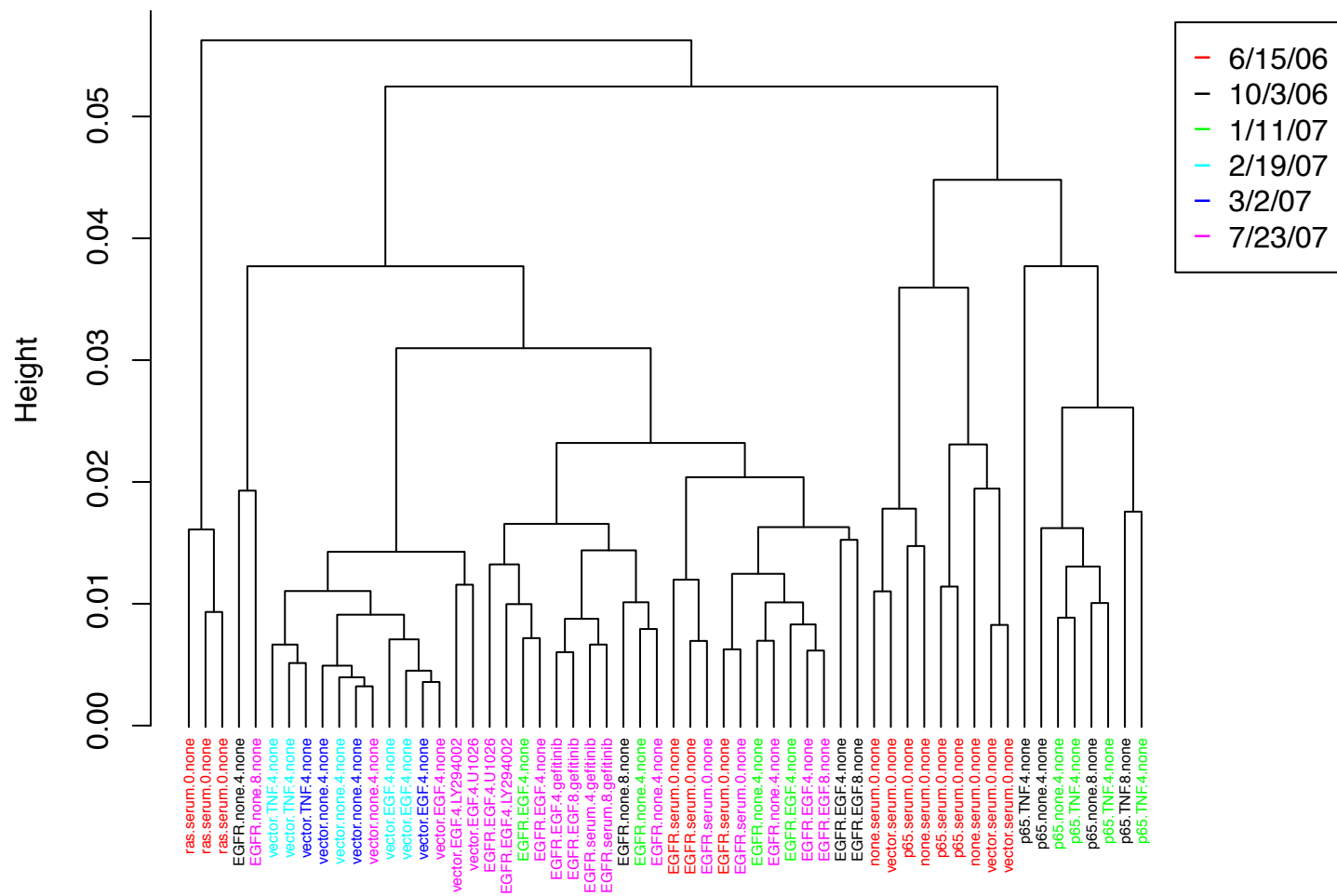

Supplement: Additional file 3 — Figure S3. Clustering of HaCaT expression data after fRMA colored by date (a). Analogous clustering after batch correction in (b). [file 1471-2164-13-160-S3.pdf]

CoGAPS Simulation 1

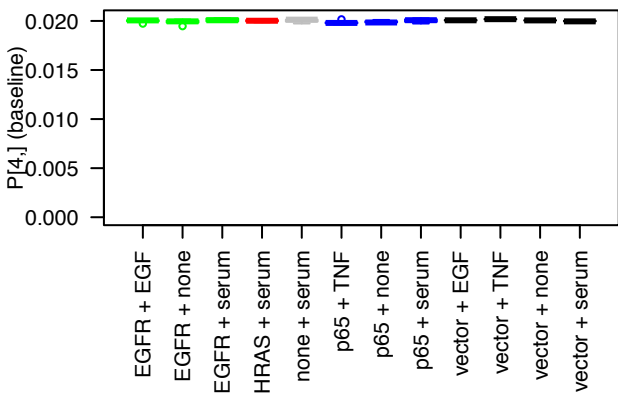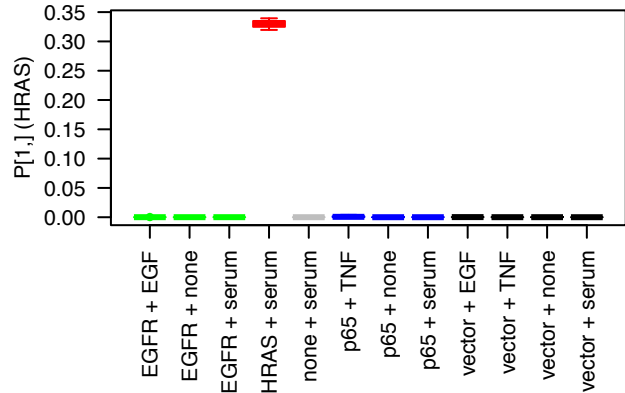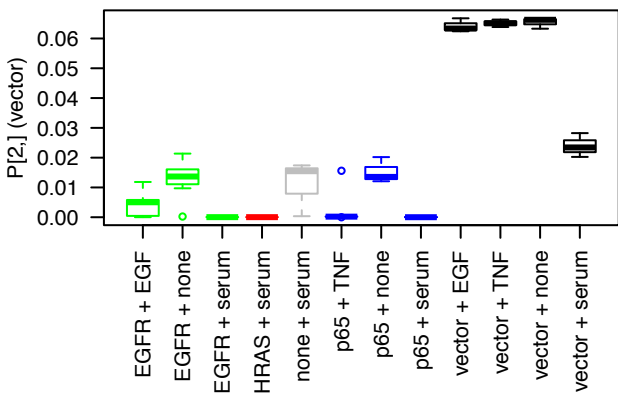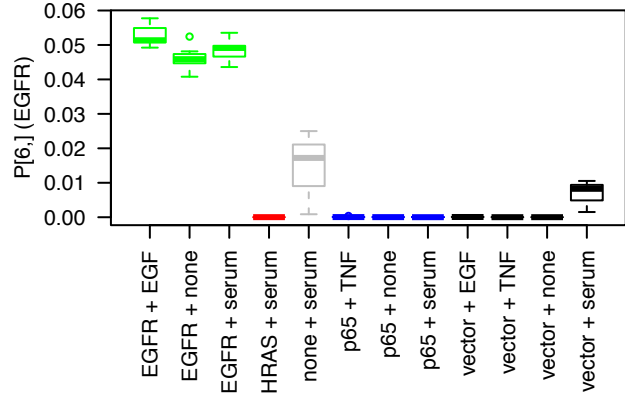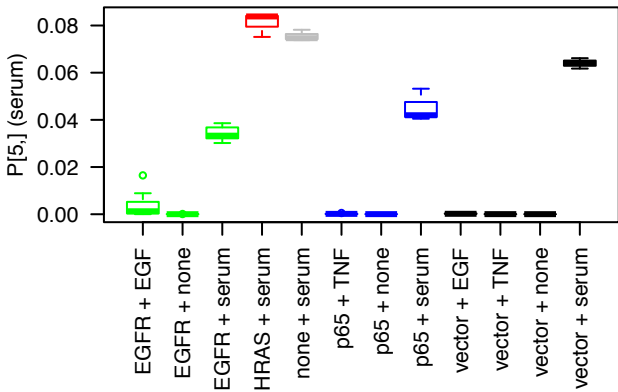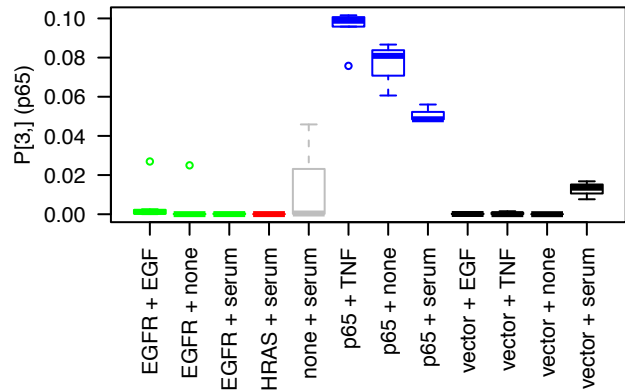

CoGAPS Simulation 2

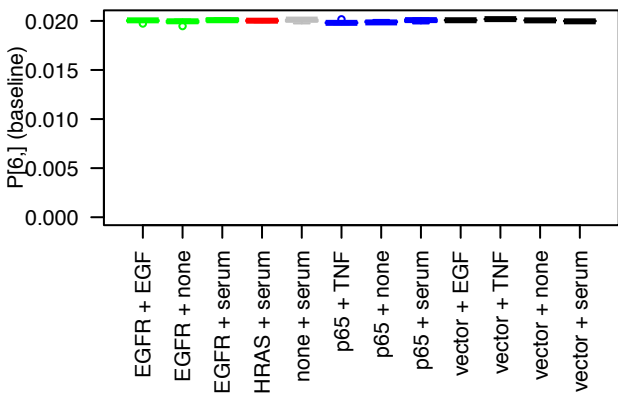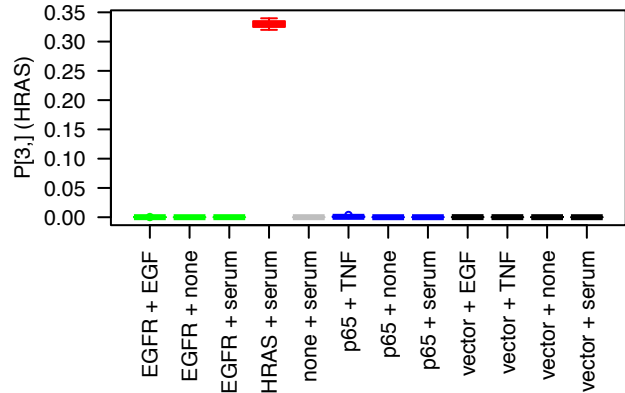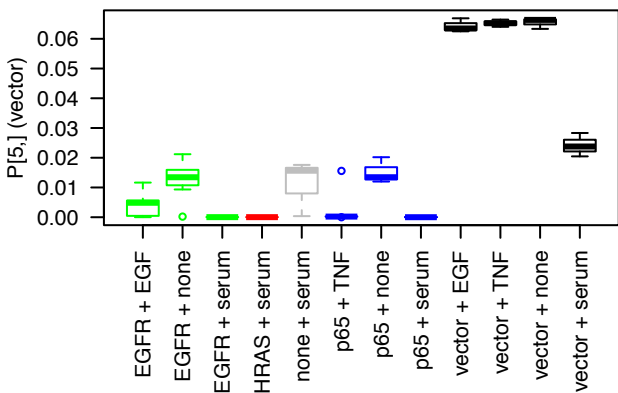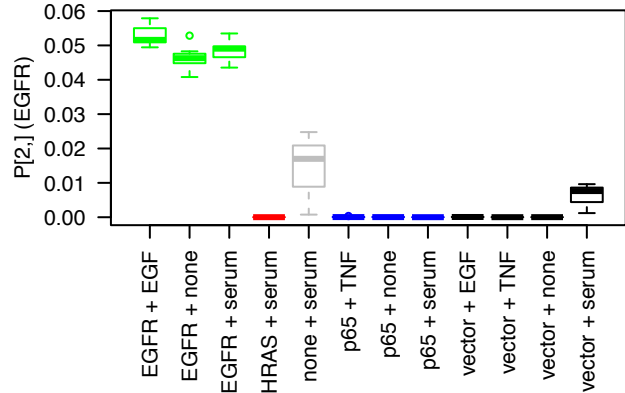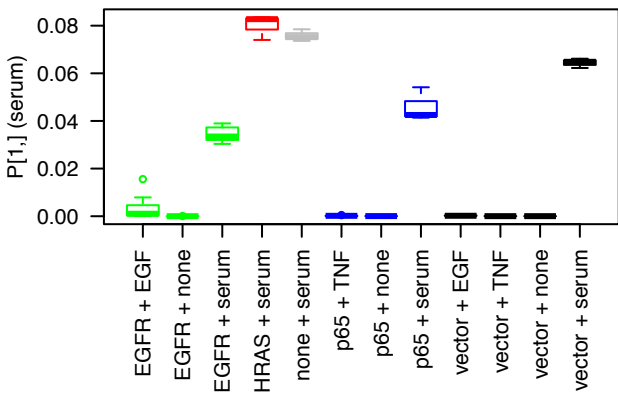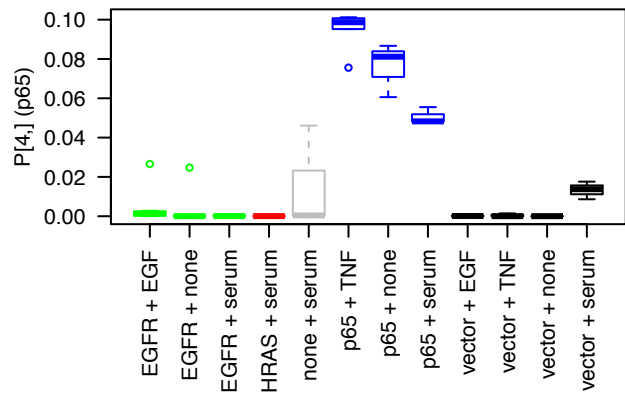

CoGAPS Simulation 3

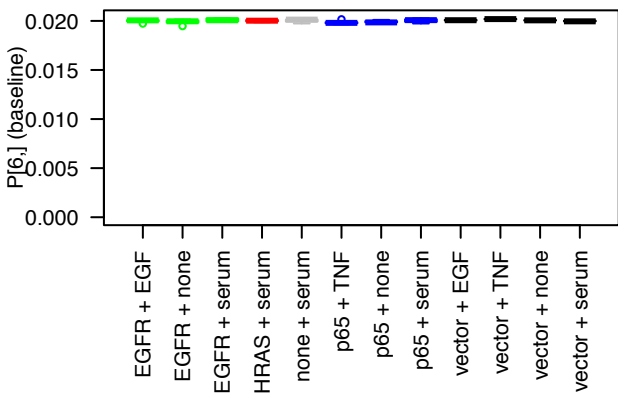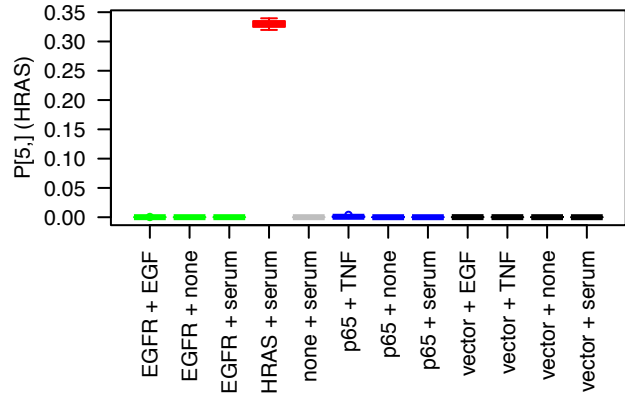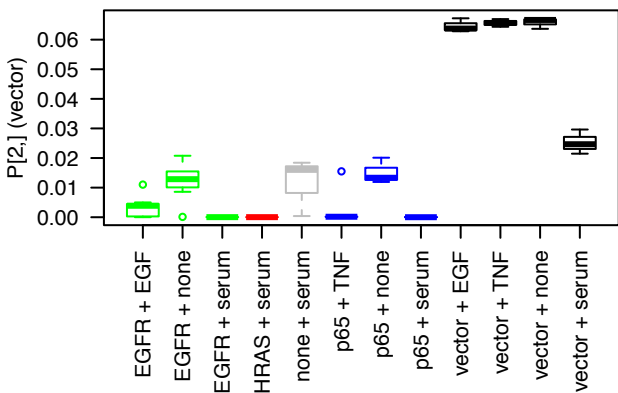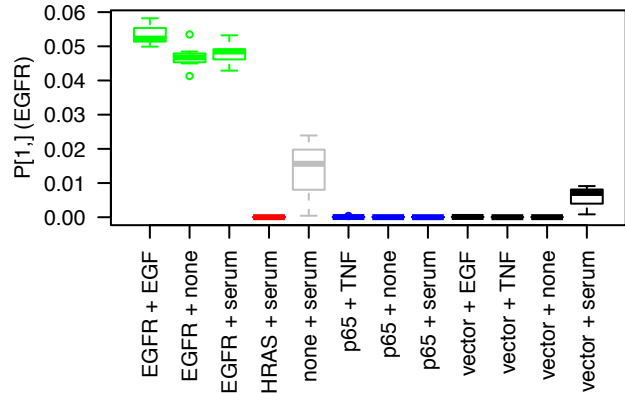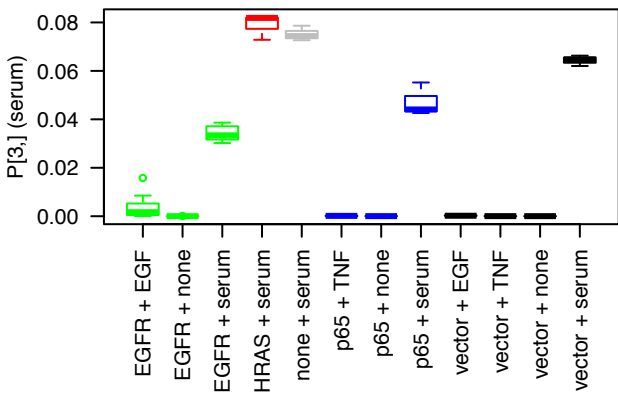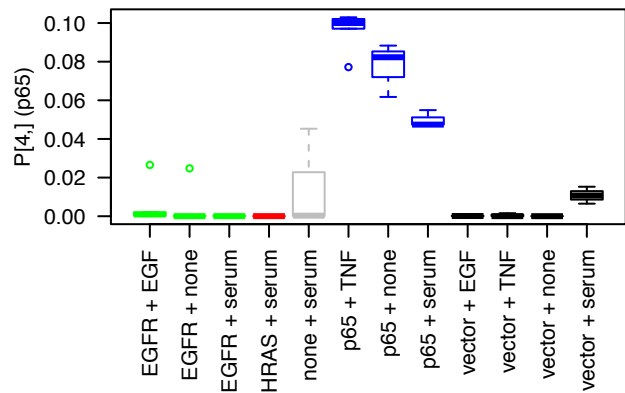

Supplement: Additional file 1 — Figure S1.Box plot of six gene expression patterns inferred from the HaCaT gene expression data for each of the three CoGAPS simulations (pages 1–3) for the samples in Table 1. Plotted values are normalized to sum to one across all samples. All results for HaCaT-EGFRWT are colored in green, HaCaT-HRASVal12D in red, HaCaT-EGFRWT in grey, HaCaT-p65WT in blue, and HaCaT-vector in black. The y-axis is labeled according to the row of the inferred P matrix plotted in each panel. Specifically, (a) contains the pattern attributed to the baseline HaCaT activity, (b) attributed to HaCaT-HRASVal12D, (c) HaCaT-vector, (d) HaCaT-EGFRWT, (e) serum, and (f) HaCaT-p65WT. [file 1471-2164-13-160-S1.pdf]

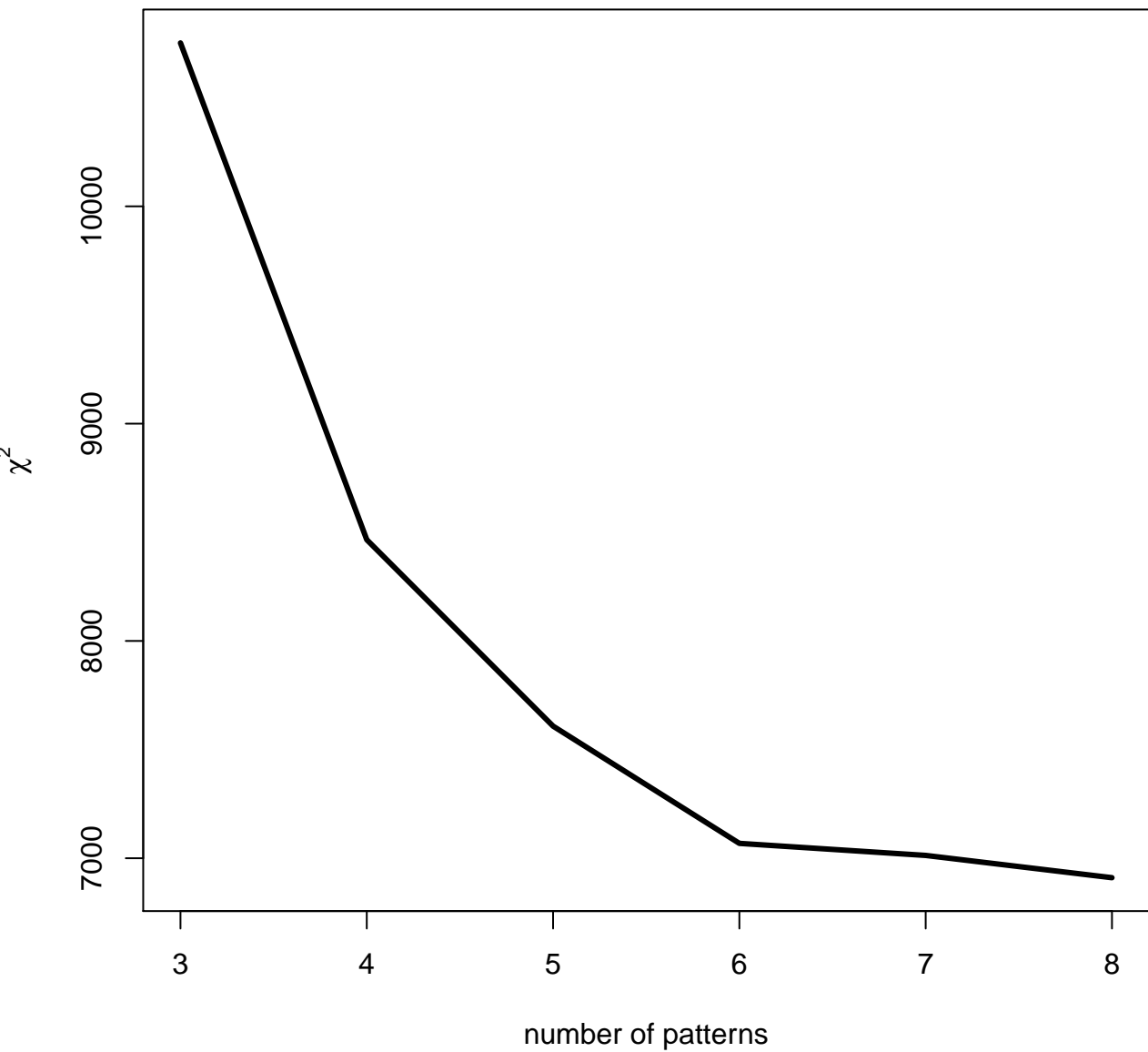

Supplement: Additional file 4 — Figure S4. χ2 fit from CoGAPS as a function of the number of patterns used in the matrix factorization for eq. 1. [file 1471-2164-13-160-S4.pdf]

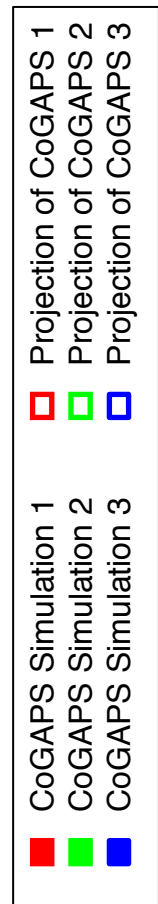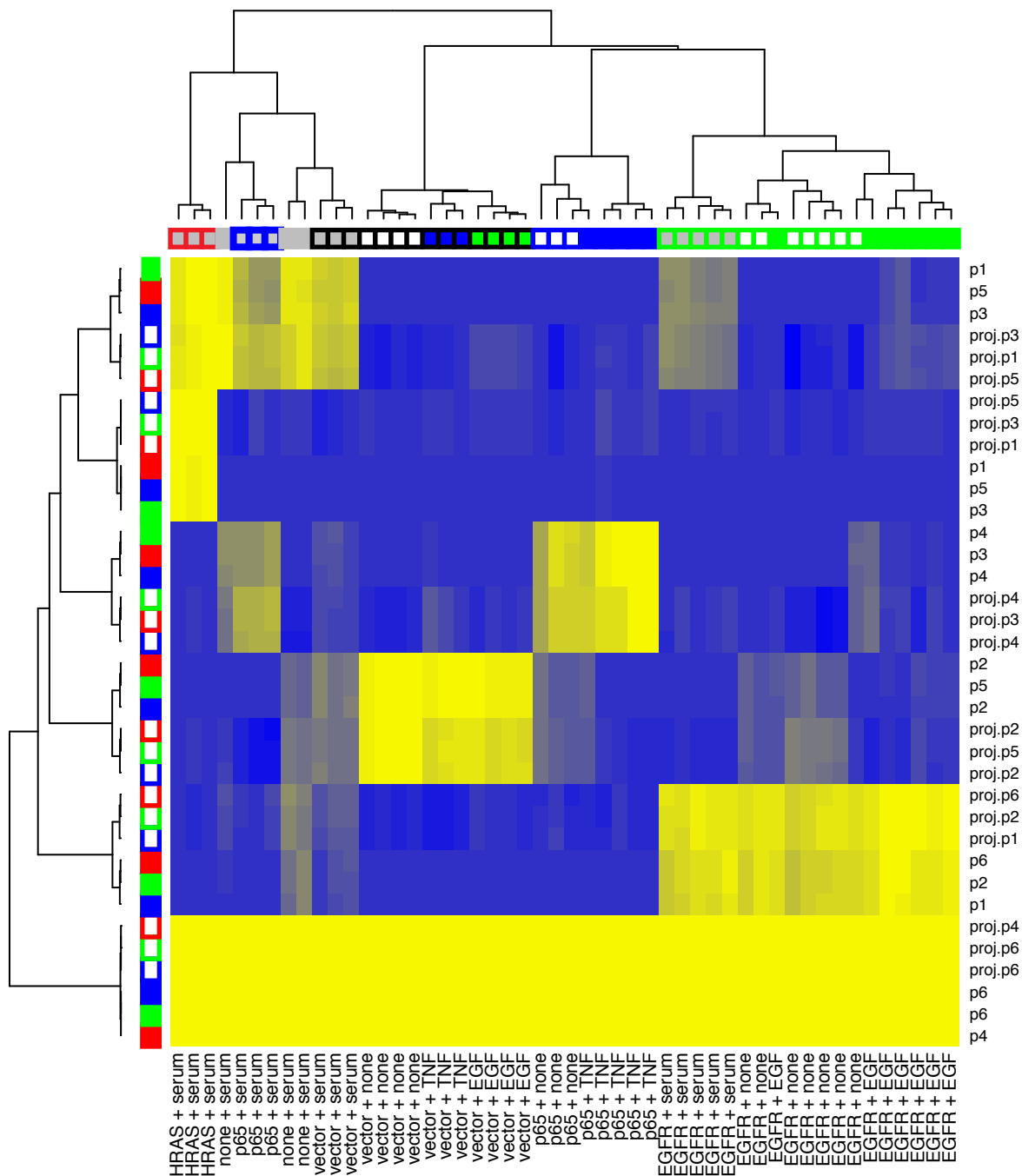

Supplement: Additional file 5 — Figure S5. Heatmap comparing patterns inferred in CoGAPS as plotted Figure S2 (filled boxes on rows) to patterns that would be inferred from projecting expression patterns as described in the methods (open boxes on rows) colored according to the row figure legend. As indicated in the row figure legend, patterns are plotted for each of three CoGAPS simulations, colored in red (simulation 1), green (simulation 2), and blue (simulation 3) along the rows. The bars across the columns indicate media and forced expression conditions, colored according to the figure legend. Shading of these bars indicates media (white for serum starved, grey for serum, green for EGF, and blue for TNFα) while borders indicate forced expression (grey for HaCaTWT, black for HaCaT-vector, green for HaCaT-EGFRWT, blue for HaCaT-p65WT, and red for HaCaT-HRASVal12D). [file 1471-2164-13-160-S5.pdf]
