# Supplementary material for: Gene expression signatures modulated by epidermal growth factor receptor activation and their relationship to cetuximab resistance in head and neck squamous cell carcinoma
Source: BMC Genomics. 2012 May 1;13:160. doi: 10.1186/1471-2164-13-160 (PMC3460736; doi:10.1186/1471-2164-13-160)
Supplement: Additional file 2 — Figure S2.Gene set statistics of the HaCaT expression data calculated from eq. 3 for each of the three CoGAPS simulations from blue for significantly downregulated to yellow for significantly upregulated according to the color bar. Columns are labeled according to the dominant experimental condition to which inferred CoGAPS patterns correspond and colored as indicated in the column color legend (red for the first CoGAPS simulation, green the second, and blue the third). The top set of statistics represents the gene set statistics computed at a pathway level. Colors along rows indicate the pathway for which activation statistics are calculated as indicated in the row color legend. The lower set of statistics represents the gene set statistics computed for the transcription factors activated by the pathway also indicated by colors in the rows associated with the pathway to which the transcription factor was assigned and indicated by the color code on the left. [file 1471-2164-13-160-S2.pdf]

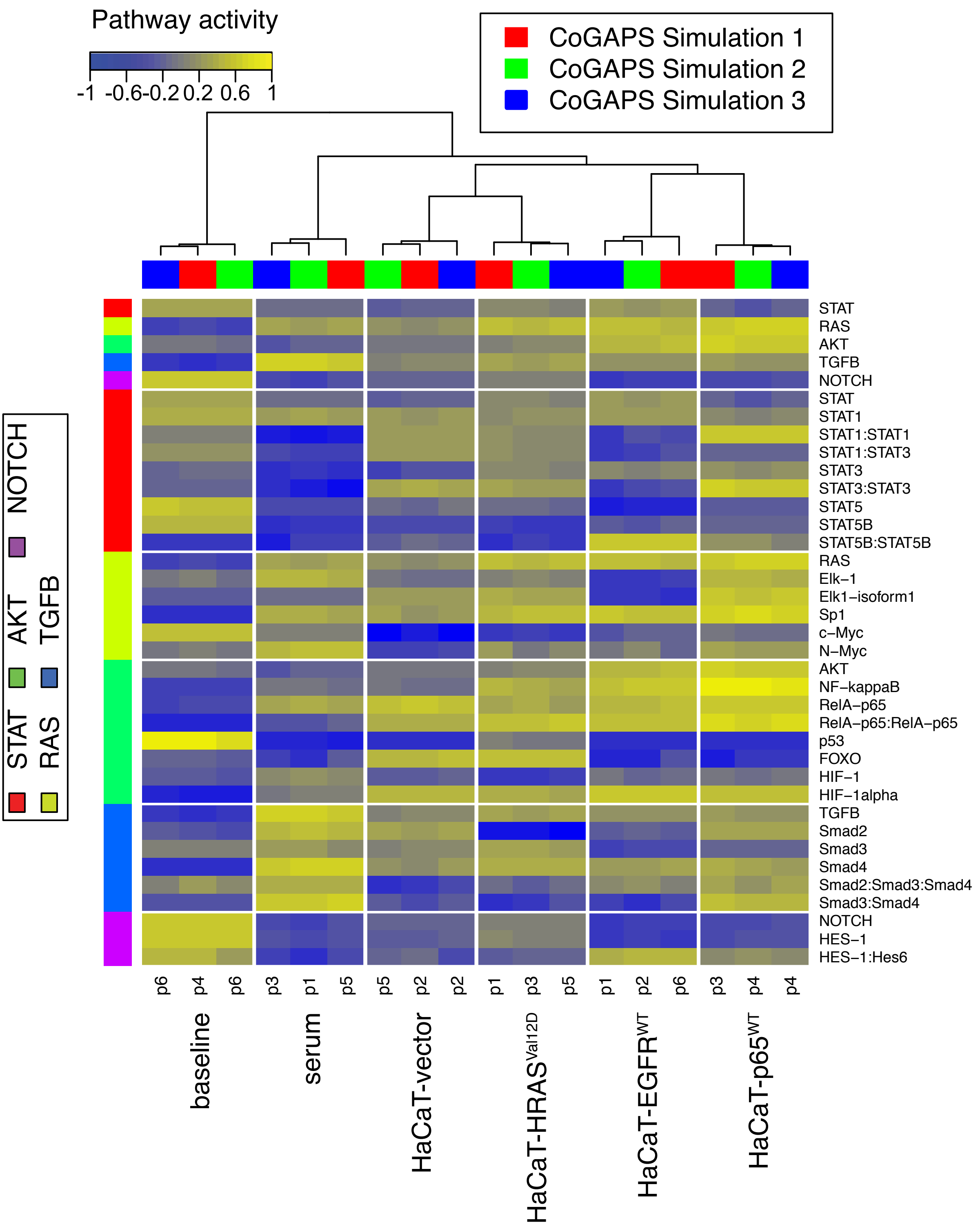
Pathway activity
-1 -0.6 -0.2 0.2 0.6 1
CoGAPS Simulation 1
CoGAPS Simulation 2
CoGAPS Simulation 3
STAT
RAS
AKT
TGFB
NOTCH
STAT
RAS
AKT
TGFB
NOTCH
STAT
STAT1
STAT1:STAT1
STAT1:STAT3
STAT3
STAT3:STAT3
STAT5
STAT5B
STAT5B:STAT5B
RAS
Elk−1
Elk1−isoform1
Sp1
c−Myc
N−Myc
AKT
NF−kappaB
RelA−p65
RelA−p65:RelA−p65
p53
FOXO
HIF−1
HIF−1alpha
TGFB
Smad2
Smad3
Smad4
Smad2:Smad3:Smad4
Smad3:Smad4
NOTCH
HES−1
HES−1:Hes6
p6 p4 p6 p3 p1 p5 p5 p2 p2 p1 p3 p5 p1 p2 p6 p3 p4 p4
baseline
serum
HaCaT-vector
HaCaT-HRAS^{Val12D}
HaCaT-EGFR^{WT}
HaCaT-p65^{WT}
